# ﻿A new species of *Opsariichthys* (Teleostei, Cypriniformes, Xenocyprididae) from Southeast China

**DOI:** 10.3897/zookeys.1214.127532

**Published:** 2024-10-01

**Authors:** Xin Peng, Jia-Jun Zhou, Hong-Di Gao, Jin-Quan Yang

**Affiliations:** 1 Shanghai Universities Key Laboratory of Marine Animal Taxonomy and Evolution, Shanghai Ocean University, Shanghai 201306, China Shanghai Ocean University Shanghai China; 2 Zhejiang Forest Resource Monitoring Center, Hangzhou 310020, China Zhejiang Forest Resource Monitoring Center Hangzhou China; 3 Zhejiang Forestry Survey Planning and Design Company Limited, Hangzhou 310020, China Zhejiang Forestry Survey Planning and Design Company Limited Hangzhou China

**Keywords:** Cytochrome *b*, morphology, opsariichthine, phylogenetic analysis, principal component analysis (PCA), taxonomy

## Abstract

*Opsariichthysiridescens***sp. nov.** is described from the Qiantang and Oujiang rivers in Zhejiang Province and a tributary of the Yangtze River adjacent to the Qiantang River. It is distinguished from congeners by the following combination of morphological features: no obvious anterior notch on the tip of the upper lip; 45–52 lateral-line scales; 18–21 pre-dorsal scales; two rows of pharyngeal teeth; a maxillary extending to or slightly beyond the vertical anterior margin of the orbit in adult males; a pectoral fin extending to the pelvic fin in adult males; nuptial tubercles on the cheeks and lower jaw of males, which are usually united basally to form a plate; uniform narrow pale pink cross-bars on trunk and two widening significantly on caudal peduncle. Its validity was also supported by its distinct Cyt *b* gene sequence divergence from all congeners and its monophyly recovered in a Cyt *b* gene-based phylogenetic analysis.

## ﻿Introduction

The genus *Opsariichthys* Bleeker, 1863, are a group of small-sized cyprinid fishes endemic to East Asia that live in fast-flowing rivers or streams (Chen 1982; Chen and Chang 2005; Wang et al. 2019). The type species, *Opsariichthysuncirostris* (Temminck & Schlegel, 1846), was initially described from Japan and assigned to the genus *Leuciscus* Cuvier, 1816. Since the genus *Opsariichthys* was first established, its earliest members, such as *Zacco* Jordan & Evermann, 1902 and *Candidia* Jordan & Richardson, 1909, that belonged to the so-called opsariichthine group and have large and elongated anal fins and a series of nuptial tubercles on the jaws as common adult features (Chen 1982), have been placed in this genus, and a total of 27 species have been described over the last one hundred years or more (Fricke et al. 2024). Chen (1982) taxonomically defined the opsariichthine fishes and noted that *Opsariichthys* included only two species, among which *O.uncirostris* was distributed in Japan and the other species, *O.bidens* Günther, 1873, was distributed in East Asia. Chen and Chu (1998) continued to follow this opinion.

Both morphological and molecular studies have shown that *Opsariichthys* and *Zacco* are closely related genera (Bǎnǎrescu 1968; Chen 1982; Huang et al. 2017; Wang et al. 2019; Zhang et al. 2023), and the traditional morphological features that distinguishes these two genera are that the former have a conspicuous notch on the tip of their upper lip and undulated jaws, while the latter has no obvious notch on the tip of their upper lip and relatively straight jaws (Jordan and Evermann 1902; Bǎnǎrescu 1968; Chen 1982; Chen and Chu 1998). However, recent molecular studies have revealed distinct genetic differentiation and multiple genetic lineages within *O.bidens* and *Z.platypus* (Temminck & Schlegel, 1846), which may correspond to different species based on traditional diagnostic features (Berrebi et al. 2005; Perdices et al. 2004; 2005). Furthermore, the genetic lineages of both species are paraphyletic (Perdices and Coelho 2006). These two species are sympatric in many places and are considered widespread in East Asia (Wang et al. 2019).

Subsequently, based on the results of morphological and phylogenetic studies, Chen et al. (2009) proposed new diagnostic key features of these two genera, suggesting that the nuptial tubercles on the cheeks of male *Opsariichthys* were separated and that the pale green lateral cross-bars were clear and independent, while the nuptial tubercles on the cheeks of male *Zacco* were united basally to form a plate and that the lateral pale green cross-bars were fused into fewer large patches. A phylogenetic study based on the mitochondrial genome by Huang et al. (2017) reaffirmed that the lateral cross-bars were a key diagnostic feature in the taxonomy of the opsariichthine group. This view has now been widely accepted. Based on this new classification, the following four new species are described: *O.songmaensis* Nguyen & Nguyen, 2000; *O.dienbienensis* Nguyen & Nguyen, 2000; *O.kaopingensis* Chen & Wu, 2009; and *O.duchuunguyeni* Huynh & Chen, 2013. Three species, viz., *O.acutipinnis* (Bleeker, 1871), *O.evolans* (Jordan & Evermann, 1902), and *O.macrolepis* (Yang & Hwang, 1964), formerly known as *Z.platypus*, are reinstated as valid *Opsariichthys* species. Three species, viz., *O.amurensis* Berg 1932, *O.minutus* Nichols, 1926, and *O.hainanensis* Nichols & Pope, 1927, that were once considered to be synonymous with *O.bidens* are also revalidated. Two species, *O.chengtui* (Kimura 1934) and *O.pachycephalus* (Günther 1868), are transferred from *Zacco* to *Opsariichthys*, while the taxonomic status of two other species, *O.bea* Nguyen 1987 and *O.hieni* Nguyen 1987, remains uncertain (Fricke et al. 2024). Therefore, *Opsariichthys* is currently considered to include 14 valid species, eight of which are distributed across mainland China. The valid species in mainland China are *O.acutipinnis*, *O.amurensis*, *O.bidens*, *O.chengtui*, *O.evolans*, *O.hainanensis*, *O.macrolepis*, and *O.minutus*, and with the exception of *O.bidens*, are all regionally distributed species.

While examining *Opsariichthys* specimens collected from Zhejiang Province and the tributaries of the Yangtze River adjacent to the Qiantang River, we found that some of the specimens did not belong to any described species. Further morphological and molecular analyses of these specimens support that they belong to a new species, which we describe here.

## ﻿Materials and methods

### ﻿Sample collection and morphological analysis

**Figure 1.**
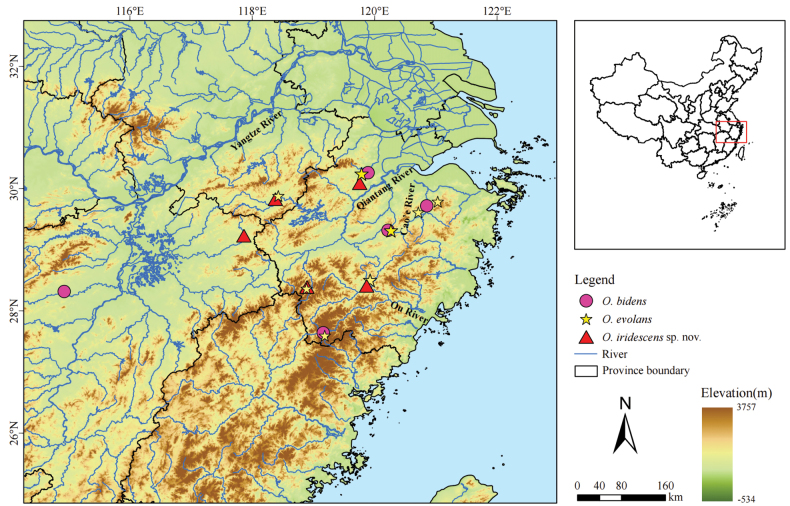
Map showing sampling sites of *O.iridescens* sp. nov. and its two sympatric species that were examined in the present study.

Sixteen specimens were collected from the Qiantang River system in Lin’an District, Hangzhou City, and Suichang County, Lishui City, Zhejiang Province, as well as from the Qiantang River region in She County, Huangshan City, Anhui Province. The right pectoral fins of these freshly collected specimens were preserved in 95% ethanol for molecular biology analyses. Meanwhile, specimens with left fins were fixed in 10% formalin for three days and then transferred to 70% ethanol for long-term preservation and subsequent morphological analyses. The specimens used in the present study were deposited at Shanghai Ocean University, Shanghai, China (SHOU). Two species (*O.bidens* and *O.evolans*) were used for deep morphological comparison with the new species because their sympatric distribution (Fig. 1). Data of other similar *Opsariichthys* species for comparison were cited from literatures (Yang and Huang 1964; Chen et al. 2009; Huynh and Chen 2013; Wang et al. 2019).

The morphometric measurements and meristic counts generally followed those of Chen et al. (2009) and Huynh and Chen (2013). Morphometric characteristics were measured with digital calipers and recorded to the nearest 0.1 mm. Counts and measurements were made on the left side of the specimen. The meristic abbreviations are as follows: D, dorsal fin; A, anal fin; P1, pectoral fin; P2, pelvic fin; LL, lateral-line scales; LLa, scales above the lateral line; LLb, scales below the lateral line; PreD, predorsal scales; and CPS, circum-peduncular scales. All the fish were measured for standard length (SL).

Based on the morphological data, principal component analysis (PCA) was performed on the three *Opsariichthys* species using R software. From the cumulative contribution of the principal components, the scores of the first principal component (PC1) and the second principal component (PC2) were plotted. Canonical discriminant analysis (CDA) and graphing were performed using SPSS version 23.0.

### ﻿DNA extraction, PCR amplification, and sequencing

Genomic DNA was extracted using an animal genomic DNA extraction kit from Shanghai Sangon Biotech Co., Ltd. The cytochrome *b* gene (Cyt b) was amplified using polymerase chain reaction (PCR) with the primers L14724 (5’-GACTTGAAAAACCACCGTTG-3’) and H15915 (5’-CTCCGATCTCCGGATTACAAGAC-3’) (Xiao et al. 2001). Each 25 μL of PCR reaction mixture contained 1 μL of DNA template, 1 μL of each primer, 12.5 μL of Taq PCR Mix (Sangon Biotech Co., Ltd., Shanghai, China), and 9.5 μL of ddH_2_O. The PCR conditions were as follows: pre-denaturation at 95 °C for 3 min; denaturation at 94 °C for 30 s; annealing at 54 °C for 40 s; extension at 72 °C for 1 min; 35 cycles of extension at 72 °C for 5 min; and heat preservation at 4 °C. After the PCR reaction was completed, the products were detected by agarose (1.5%) electrophoresis and sequenced bidirectionally by Shanghai Sangon Biotech Co., Ltd. The sequencing results for the Cyt *b* sequences were manually corrected and assembled using SeqMan software from DNASTAR (Burland 2000).

### ﻿Phylogenetic analysis

A total of 74 sequences were used, 45 of which were newly sequenced and 29 of which were obtained from GenBank. The specific sample information is shown in Table 1. MEGA v. 11.0 (Tamura et al. 2021) was used to align the sequences and calculate the nucleotide composition, variable sites, parsimony informative sites and genetic distances between species. Neighbor joining (NJ) analysis was also performed with MEGA v. 11.0 using the Kimura 2-parameter (K2P) model. Bootstrapping with 1,000 pseudo replicates was used to examine the robustness of the clades in the resulting tree. The best substitution model (TIM2+I+G) was selected for maximum likelihood (ML) analysis and Bayesian inference (BI) analysis using jModeltest v. 2.0 software (Darriba et al. 2012) with the Akaike information criterion (AIC). ML analysis was conducted using IQ-TREE v. 2.0 software (Minh et al. 2020), and node confidence was analyzed by bootstrap analysis with 1,000 repetitive samples. Mrbayes v. 3.2.6 software (Ronquist et al. 2012) was used to conduct the BI analysis, and posterior probability was used to indicate the credibility of each branch. The starting tree was set as a random tree. Four Markov chains were run simultaneously for two million generations, with three hot chains and one cold chain. The system tree was sampled every 100 generations to remove the top 25% of untrustworthy regions, and the process was stopped when the variance of convergence was less than 0.01. All trees were viewed and edited using FigTree v. 1.4.3 software (Rambaut 2016).

**Table 1.** The samples used in this study with their localities, voucher information, and GenBank accession numbers.

Genus	Species	Location	River	Voucher number	GenBank accession number
*Opsariichthys*	*O.iridescens* sp. nov.	Lin’an, Zhejiang	Qiantang River	ZJQT01-05	PP639122–PP639123, PP639130–PP639132*
		Huangshan, Anhui	Qiantang River	ZJXA01-03	PP639124–PP639126*
		Wuyuan, Jiangxi	Yangtze River	ZJLA01-03	PP639127–PP639129*
		Lishui, Zhejiang	Ou River	ZJOJ01-03	PP639133–PP639135*
	*O.bidens*	Lin’an, Zhejiang	Qiantang River	MKQT01-02	PP639101–PP639102*
		Dongyang, Zhejiang	Qiantang River	MKQT03	PP639103*
		Shengzhou, Zhejiang	Cao’e River	MKCE	PP639097*
		Yichun, Jiangxi	Gan River	MKJJ01-03	PP639098–PP639100*
		Fujian	Jiulong River	OBJLJ1-2	FJ602005–FJ602006
	*O.evolans*	Lin’an, Zhejiang	Qiantang River	CQQT01-02	PP639110–PP639111*
		Dongyang, Zhejiang	Qiantang River	CQQT03-07	PP639112–PP639116*
		Quzhou, Zhejiang	Qiantang River	ZP_QTJ_1-2	MH350437–MH350438
		Shangyu, Zhejiang	Cao’e River	CQCE01	PP639104*
		Shengzhou, Zhejiang	Cao’e River	CQCE02-06	PP639105–PP639109*
		Lishui, Zhejiang	Ou River	CQOJ01-02	PP639117–PP639118*
		Taiwan	Unknown	OETaiW1-2	KR698567–KR698568
	*O.macrolepis*	Hejiang, Sichuan	Yangtze River	ZP_CJU2_1	MH350702
	*O.hainanensis*	Hainan	Unknown	OHAND2	KJ940933
	*O.chengtui*	Chengdu, Sichuan	Yangtze River	–	KT725244
	*O.acutipinnis*	Huangshan, Anhui	Yangtze River	ZPQimen4	KM491719
	*O.duchuunguyeni*	Baise, Guangxi	Pear River	ZPPE_You1	KP101024
	*O.pachycephalus*	Taiwan	Unknown	OPTaiW1	KR698649
	*O.kaopingensis*	Taiwan	Unknown	–	AY958189
	*O.minutus*	Guangxi	Pear River	OMhap01	KR698540
	*O.uncirostris*	Japan	Unknown	OUJapan1	KR698682
	*Opsariichthys* sp. A	Huangshan, Anhui	Yangtze River	ZPTaiping2	KM491721
	*Opsariichthys* sp. B	Fujian	Min River	OEMinJ1	KR698572
	*Opsariichthys* sp. C	Hunan	Yangtze River	OEXiangJ5	KR698575
	*Opsariichthys* sp. D	Jiangxi	Yangtze River	ZA_FH2	MH350668
	*Opsariichthys* sp. E	Hunan	Yangtze River	OELI1	KR698563
*Zacco*	*Z.acanthogenys*	Shengzhou, Zhejiag	Qiantang River	JJQT01-03	PP639119–PP639121*
	*Z.tiaoxiensis*	Yuhang, Zhejiang	Tiaoxi River	TX01-03	PP639136–PP639138*
	*Z.sinensis*	Fengcheng, Liaoning	Yalu River	ZHYL01-03	PP639139–PP639141*
	*Z.platypus*	Japan	Miya River	ZPWJ1	LC019793
*Parazacco*	*P.spilurus*	Unknown	Unknown	PS1	KF971863
	*P.fasciatus*	Unknown	Unknown	PF1	AY958195
*Nipponocypris*	*N.temminckii*	Unknown	Unknown	NT1	EF452750
	*N.sieboldii*	Unknown	Unknown	NS1	AY958198
*Candidia*	*C.barbatus*	Taiwan	Fenggang River	CB1	AY958200
	*C.pingtungensis*	Taiwan	Gaoping River	CP1	KT725246
*Aphyocypris*	*A.chinensis*	Japan	Unknown	–	NC008650
	*A.chinensis*	China	Unknown	–	AF307452

Note: * Sequenced in this study.

## ﻿Results

### ﻿Taxonomic account

**Family Xenocyprididae Günther 1868**

**Genus *Opsariichthys* Bleeker, 1863**

#### ﻿ Opsariichthys iridescens

Taxon classificationAnimaliaCypriniformesCyprinidae

Peng, Zhou & Yang sp. nov.

85E27CFC-0FE3-5B87-AC4C-5177E045AC59

https://zoobank.org/FADF08EA-9DBC-45A3-9D67-79274C1B3083

Figs 2 , 3A, B

##### Type material.

***Holotype*** • SHOU202210001, male, adult, 91.0 mm standard length (SL), collected by Jia-Jun Zhou and Hui Cao in October 2022, in Lin’an District, Hangzhou City, Zhejiang Province (Qiantang River) (30.2368°N, 119.7196°E). ***Paratypes*** • SHOU202210002–SHOU202210010, 9 specimens, 79.1–96.0 mm standard length (SL), collected by Jia-Jun Zhou and Hui Cao in October 2022, from the same locality as the holotype; SHOU202106089, SHOU202106090, and SHOU202106125, 3 specimens, 85.7~110.7 mm standard length (SL), collected by Jia-Jun Zhou and Wei Sun in June 2021, in Suichang County, Lishui City, Zhejiang Province (Qiantang River) (28.5956°N, 119.2709°E); SHOU202106001–SHOU202106003, 3 specimens, 84.5~109.4 mm standard length (SL), collected by Yun-Feng Huang in June 2021, in Shexian County, Huangshan City, Anhui Province (Qiantang River) (29.8637°N, 118.4100°E).

##### Diagnosis.

The new species, *Opsariichthysiridescens* sp. nov. can be clearly distinguished from its two sympatric congeners in the Qiantang River and nearby geographic regions (Tables 3, 4). It can be distinguished from *O.evolans* by the following features: (1) lateral-line scales 45–52 (vs 42–45); (2) scales above lateral-line nine or ten (vs 8); (3) pre-dorsal scales 18–21 (vs 15–17); (4) two rows of pharyngeal teeth (vs 3 rows); (5) maxillary extending to or slightly beyond the vertical of anterior margin of orbit in adult male (vs never extending to the vertical of anterior margin of orbit); (6) pectoral fin extending to pelvic fin in adult male (vs extending far beyond origin of ventral fin); (7) almost uniform narrow pale cross-bars on trunk and widening significantly on caudal peduncle (vs gradually widened, Fig. 3E, F); (8) lower jaw with one row of large tubercles usually united basally to form a plate in male (vs 1 row of moderate tubercles well separated). The new species can be clearly distinguished from *O.bidens* by the following features: (1) absence of distinct anterior notch on upper lip (vs presence of conspicuous anterior notch on upper lip); (2) two rows of pharyngeal teeth (vs 3 rows); (3) maxillary extending to or slightly beyond the vertical of anterior margin of orbit in adult male (vs extending to the vertical midpoint of the eye); (4) pectoral fin extending to pelvic fin in adult male (vs never extending); (5) almost uniform narrow pale cross-bars on trunk and widening significantly on caudal peduncle (vs gradually widened, Fig. 3C, D); (6) one row of large tubercles under lower jaw united basally to form a plate in male (vs 3 or 4 rows of moderate tubercles well separated).

*Opsariichthysiridescens* sp. nov. can be well separated from the congeners: *O.uncirostris* from Japan and Korea; *O.amurensis*, *O.minutus*, and *O.hainanensis* from mainland China; *O.dienbienensis* and *O.songmaensis* from Vietnam, like *O.bidens*, by the absence of distinct anterior notch on upper lip (vs the presence of distinct anterior deep notch on that). Besides *O.evolans*, the new species can be distinguished from the remaining congeneric species: *O.acutipinnis*, *O.chengtui*, and *O.macrolepis* from mainland China; *O.kaopingensis* and *O.pachycephalus* from Taiwan; *O.duchuunguyeni* from Vietnam, that have an absence of distinct anterior notch on upper lip as well as by the following combination of morphological features (Table 4): (1) lateral-line scales 45–52; (2) scales above lateral line nine or ten; (3) pre-dorsal scales 18–21; (4) circum-peduncular scales 16 or 17; (5) two rows of pharyngeal teeth; (6) maxillary extending to or slightly beyond vertical of anterior margin of orbit in adult male; (7) pectoral fin extending to pelvic fin in adult male; (8) almost uniform, narrow, pale cross-bars on trunk, widening significantly on caudal peduncle; (9) nuptial tubercles on cheeks and lower jaw united basally to form a plate in adult male.

##### Description.

**Figure 2.**
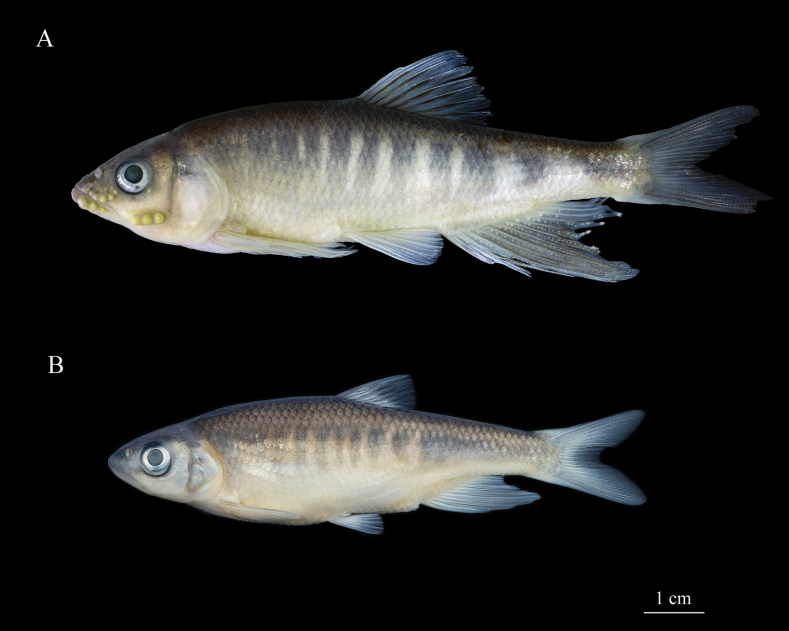
*Opsariichthysiridescens* sp. nov. **A** holotype, SHOU202210001, preserved male specimen, 91.0 mm SL**B** paratype, SHOU202308012, preserved female specimen, 85.2 mm SL.

The morphometric and meristic data are listed in Tables 2, 3. Fig. 2A, B shows lateral views of the male and female fish.

**Figure 3.**
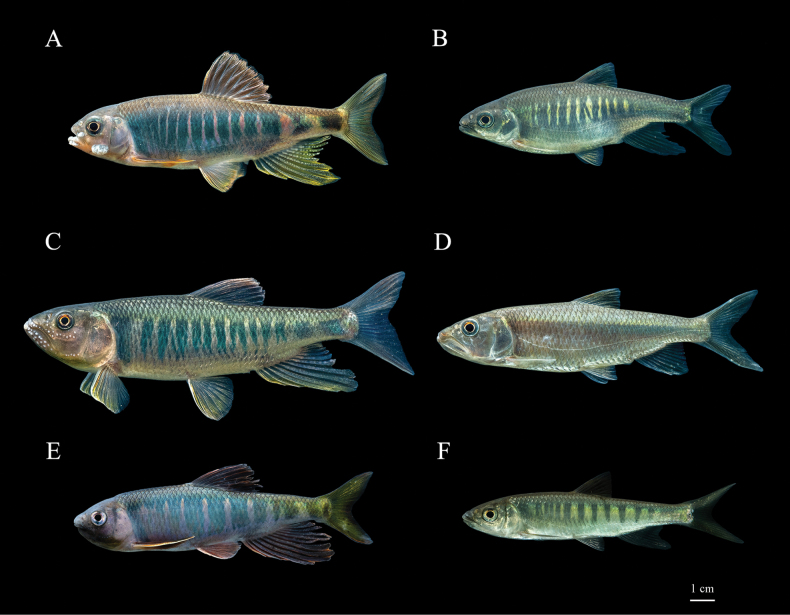
*Opsariichthysiridescens* sp. nov. **A** live male **B** live female; *Opsariichthysbidens***C** live male **D** live female; *Opsariichthysevolans***E** live male **F** live female.

**Table 2.** Morphometric measurements of *Opsariichthysbidens*, *O.evolans*, and *O.iridescens* sp. nov.

	*O.bidens*						*O.evolans*						*O.iridescens* sp. nov.						
	Male			Female			Male			Female			Holotype Male	Male			Female		
*n*	2			8			9			8			1	13			2		
Standard length (mm)	95.2~100.6			76.1~131.6			71.2~111.6			67.5~84.0			91.0	79.1~110.7			84.5~92.4		
% of SL	Min	Max	Mean	Min	Max	Mean	Min	Max	Mean	Min	Max	Mean		Min	Max	Mean	Min	Max	Mean
Body depth	24.6	26.8	25.7	20.1	25.2	22.5	22.1	25.5	24.2	22.0	24.8	23.8	24.0	23.7	28.6	26.0	23.3	24.7	24.0
Head length	30.0	30.1	30.0	29.8	31.1	30.5	23.4	26.5	24.8	24.2	26.6	25.3	27.2	25.8	27.9	27.1	26.5	27.2	26.9
Length of the caudal fin peduncle	17.3	18.2	17.7	14.6	20.0	16.5	16.3	19.3	17.6	16.2	19.5	17.9	18.5	17.1	20.1	18.7	17.6	18.1	17.9
Depth of the caudal fin peduncle	9.0	9.5	9.2	8.2	9.9	8.8	7.7	9.4	8.7	8.2	9.3	8.8	9.2	8.5	10.0	9.3	8.5	8.8	8.7
Dorsal fin length	18.7	18.9	18.8	14.1	17.4	15.8	19.3	26.0	23.3	17.8	24.9	20.9	17.2	17.0	19.8	18.2	16.5	16.8	16.7
Pectoral fin length	19.6	22.0	20.8	12.9	19.8	17.9	24.6	30.9	27.3	19.4	28.5	24.2	23.1	21.1	26.7	23.5	18.4	19.1	18.8
Pelvic fin length	15.3	16.2	15.7	11.7	14.8	13.6	16.8	22.2	19.3	13.9	19.7	17.3	15.5	14.2	17.8	15.5	12.8	14.5	13.7
Anal fin length	25.6	28.5	27.0	18.3	25.1	21.8	33.3	42.4	38.2	22.6	39.7	31.5	29.3	28.0	35.7	32.1	25.7	25.8	25.8
Dorsal fin base length	13.0	13.3	13.2	9.3	11.4	10.6	12.1	15.2	13.5	10.0	14.9	12.1	12.3	11.6	13.4	12.5	10.2	10.7	10.5
Pectoral fin base length	4.5	5.1	4.8	3.3	4.4	3.9	4.6	5.9	5.3	4.0	5.1	4.6	5.1	5.0	6.3	5.8	4.0	4.2	4.1
Pelvic fin base length	3.7	3.8	3.7	3.2	4.0	3.6	3.3	4.5	3.8	3.2	4.6	3.8	4.9	3.8	4.9	4.2	3.6	3.7	3.7
Anal fin base length	15.1	15.4	15.3	10.5	12.0	11.0	15.6	18.9	17.2	13.6	19.1	15.6	17.6	15.2	18.6	16.9	12.6	13.2	12.9
Predorsal length	53.2	53.3	53.2	51.8	56.2	53.8	48.4	50.0	48.9	48.3	50.5	49.2	51.1	49.0	55.2	51.9	51.3	54.0	52.7
Prepectoral length	26.8	27.0	26.9	27.5	29.6	28.9	23.6	25.7	24.5	23.9	27.7	25.0	24.5	24.5	26.8	25.6	26.5	26.8	26.7
Prepelvic length	50.1	52.0	51.1	51.6	54.8	53.0	46.1	49.5	47.3	46.4	50.2	48.3	46.3	46.3	49.9	48.0	49.8	50.0	49.9
Preanal length	68.9	69.3	69.1	71.2	73.9	72.6	46.6	68.6	65.1	66.6	71.5	69.1	63.1	63.1	70.4	66.2	69.4	71.2	70.3
% **of HL**																			
Snout length	29.3	32.1	30.7	29.6	33.7	32.1	25.7	33.4	29.0	27.6	31.5	29.3	31.4	28.8	35.6	31.5	29.7	30.8	30.3
Eye diameter	18.2	19.6	18.9	16.0	22.4	19.1	21.9	30.5	27.0	25.5	28.4	27.5	20.4	20.4	29.3	25.4	26.5	27.8	27.2
Interorbital width	31.7	31.9	31.8	28.2	31.4	29.7	29.5	34.7	31.4	26.1	34.4	30.7	34.3	32.0	37.1	34.8	30.9	32.5	31.7
Head depth	64.0	66.2	65.1	56.4	62.6	59.4	69.7	78.7	74.5	64.1	76.5	68.6	69.5	67.2	75.6	70.6	66.2	67.0	66.6
Head width	49.0	54.3	51.6	38.9	49.5	43.9	44.5	52.3	49.5	42.1	54.1	49.0	48.5	45.4	55.8	51.0	49.1	52.9	51.0

**Table 3.** Meristic counts of the three sympatric *Opsariichthys* species and its congeners that absence of distinct anterior notch on upper lip.

**Species**	**D iii**				**A iii**					**P1 i**					**P2 i**		
	**7**	**8**	**M**		**8**	**9**	**10**	**M**		**13**	**14**	**15**	**M**		**7**	**8**	**M**
*O.iridescens* sp. nov.	16	–	7.0		–	16	–	9.0		1	15	–	13.9		2	14	7.9
*O.bidens*	10	–	7.0		1	9	–	8.9		–	10	–	14.0		–	10	8.0
*O.evolans*	17	–	7.0		–	16	1	9.1		1	15	1	14.0		3	14	7.8
O.acutipinnis*	9	–	7.0		–	9	–	9.0		–	1	8	14.9		–	9	8.0
O.duchuunguyeni*	5	–	7.0		–	5	–	9.0		–	4	1	14.2		1	4	7.9
O.kaopingensis*	118	–	7.0		3	115	–	9.0		5	147	72	14.3		210	14	8.1
O.macrolepis*	30	–	7.0		–	30	–	9.0		–	30	–	14.0		15	15	7.5
O.pachycephalus*	421	2	7.0		17	398	12	9.0		251	251	38	13.6		251	123	8.3
	**CPS**							**LLa**						**LLb**			
	**16**	**17**	**18**	**19**	**20**	**M**		**8**	**9**	**10**	**11**	**M**		**3**	**4**	**5**	**M**
*O.iridescens* sp. nov.	5	11	–	–	–	16.7		–	7	9	–	9.6		3	13	–	3.8
*O.bidens*	–	2	7	1	–	17.9		–	10	–	–	9.0		3	6	1	3.8
*O.evolans*	10	7	–	–	–	16.4		17	–	–	–	8.0		3	14	–	3.8
O.acutipinnis*	–	–	2	6	1	18.9		2	7	–	–	8.8		1	8	–	3.9
O.duchuunguyeni*	–	4	1	–	–	17.2		5	–	–	–	8.0		5	–	–	3.0
O.kaopingensis*	–	1	1	1	1	18.5		–	95	17	–	9.2		104	7	–	3.1
O.macrolepis*	–	15	15	–	–	17.5		30	–	–	–	8.0		30	–	–	3.0
O.pachycephalus*	–	1	4	3	5	18.9		–	–	251	38	10.1		251	20	–	3.1
	**PreD**																
	**13**	**14**	**15**	**16**	**17**	**18**	**19**	**20**	**21**	**22**	**23**	**M**					
*O.iridescens* sp. nov.	–	–	–	–	–	2	5	7	2	–	–	19.6					
*O.bidens*	–	–	–	–	–	–	3	5	2	–	–	19.9					
*O.evolans*	–	–	2	10	5	–	–	–	–	–	–	16.2					
O.acutipinnis*	–	–	1	5	3	–	–	–	–	–	–	16.2					
O.duchuunguyeni*	1	4	–	–	–	–	–	–	–	–	–	13.8					
O.kaopingensis*	–	–	–	–	7	47	60	–	–	–	–	18.6					
O.macrolepis*	–	–	–	–	12	9	9	–	–	–	–	17.9					
O.pachycephalus*	–	–	–	–	–	–	–	90	139	89	25	21.1					
	**LL**																
	**41**	**42**	**43**	**44**	**45**	**46**	**47**	**48**	**49**	**50**	**51**	**52**	**53**	**54**	**M**		
*O.iridescens* sp. nov.	–	–	–	–	1	2	1	4	4	1	1	2	–	–	48.6		
*O.bidens*	–	–	–	–	2	6	2	–	–	–	–	–	–	–	46.0		
*O.evolans*	–	1	7	3	6	–	–	–	–	–	–	–	–	–	43.8		
O.acutipinnis*	–	7	2	–	–	–	–	–	–	–	–	–	–	–	42.2		
O.duchuunguyeni*	5	–	–	–	–	–	–	–	–	–	–	–	–	–	41.0		
O.kaopingensis*	–	–	–	13	84	66	57	2	–	–	–	–	–	–	45.8		
O.macrolepis*	–	–	–	–	–	15	10	4	1	–	–	–	–	–	46.7		
O.pachycephalus*	–	–	–	–	–	–	–	2	59	121	129	137	120	108	51.7		

M: mean of all listed values; *: data cited from the literature.

**Table 4.** Morphological differences among eight *Opsariichthys* species that absence of distinct anterior notch on upper lip.

Character	*O.iridescens* sp. nov.	*O.evolans*	O.acutipinnis*	O.chengtui*	O.duchuunguyeni*	O.kaopingensis*	O.macrolepis*	O.pachycephalus*
Lateral-line scales	45–52	42–45	42–43	60–67	41	44–48	46–49	48–54
Scales above the lateral line	9–10	8	8–9	11	8	9–10	8	10–11
Predorsal scales	18–21	15–17	15–17	25–26	13–14	17–19	17–19	20–23
Circum-peduncular scales	16–17	16–17	18–20	21–22	17	17–20	17–18	17–20
Pharyngeal teeth	2 rows	3 rows	3 rows	2 rows	3 rows	3 rows	2 rows	3 rows
Whether the maxillary extending the vertical of anterior margin of orbit	Extending to or slightly beyond	Not reaching to or slightly extending	Extending	Extending	Extending to or slightly beyond	Reaching or slightly beyond	Not reaching	Extending to or beyond the middle vertical of orbit
Whether the pectoral extends to the origin of the pelvic fin	Slightly extending	Extending far beyond	Never reaching	Never reaching	Not reaching or slightly extending	Never reaching	Not reaching or slightly extending	Never reaching
Features of the bright bars on the flanks	Uniform and narrow on the trunk and widening significantly on the caudal peduncle	Gradually widened	Gradually widened	Gradually widened	Gradually widened	Uniform on the trunk and wider on the caudal peduncle	Gradually widened	Gradually widened
The number of tubercles on the lower jaw of adult males	1 row, united basally to form a plate in males	1 row, well separated	1 row, well separated	1 row, well separated	2 rows, well separated	1 row, well separated	2 or 3 rows, well separated	1 row, well separated

*: data cited from literature.

Dorsal fin rays iii, 7; anal fin rays iii, 9; pectoral fin rays i,13–14; pelvic fin rays i,7–8; lateral-line scales 45–52; scales above lateral line nine or ten; scales below lateral line three or four; predorsal scales 18–21; circum-peduncular scales 16 or 17; and two rows of pharyngeal teeth.

Body elongated and laterally compressed, belly rounded. Body depth slightly shorter than head length. No maxillary or rostral barbels. Mouth subterminal and oblique, maxillary extending to or slightly beyond the vertical of anterior margin of orbit. Mouth lacking obvious anterior notch and jaws relatively straight. Eyes rather large, upper lateral. Interorbital width approximately equal to or slightly less than snout length. Distinct nuptial tubercles on head and anal fin rays of adult male, one row of 3–6 on each side of lower jaw, one row of three or four on cheek. One row of 4–6 large, rounded tubercles on snout, usually united basally to form a plate. Body with moderately cycloid scales. Lateral line complete, depressed downward above pectoral fin and extending along lower half of body to mid-lateral on caudal peduncle. Tiny scales on belly.

Pectoral fin reaching or extending slightly beyond pelvic fin origin when depressed in adult male, but not reaching the origin in female. Pelvic fin origin vertical or slightly behind dorsal fin origin, extending to anal fin origin when depressed in adult male, but not reaching the origin in female. Anal fin rays rather elongate, especially first to fourth branched rays longer in male, with the rear tip extending beyond vertical line of caudal fin base. Caudal fin forked, lower lobe almost equal to upper one.

***Coloration.*** In life, body brightly colored, males more colorful than females. Ten to thirteen irregular blue-green cross-bars separated by pale cross-bars on the flanks. In adult male, uniform narrow pale pink cross-bars on trunk and two on caudal peduncle widening significantly; upper and lateral sides of head grayish and transitioning to orange-red on ventral side and lower margin of cheek. Dorsal fin rays transparent and membrane black-grey with orange margin. Anal fin and caudal fin rays transparent, membrane pale yellow or colorless. Pectoral fins orange and pelvic fins yellow (Fig. 3A). In females, narrow bright yellow bars on trunk and always absent on caudal peduncle. Dorsal fin black-grey. Pectoral fin orange yellow. Pelvic fin, anal fin and caudal fin transparent and colorless (Fig. 3B). In 10% formalin-fixed specimens, dorsal and flank of head and body grayish brown; ventral surface of head and abdomen white to yellowish. Dorsal and caudal fin dark gray. Pectoral, pelvic and caudal fin grayish white (Fig. 2).

##### Distribution.

The new species is only found in Qiantang and Oujiang River systems in Zhejiang Province and the tributaries of the lower Yangtze River adjacent to the Qiantang River.

##### Habitat.

**Figure 4.**
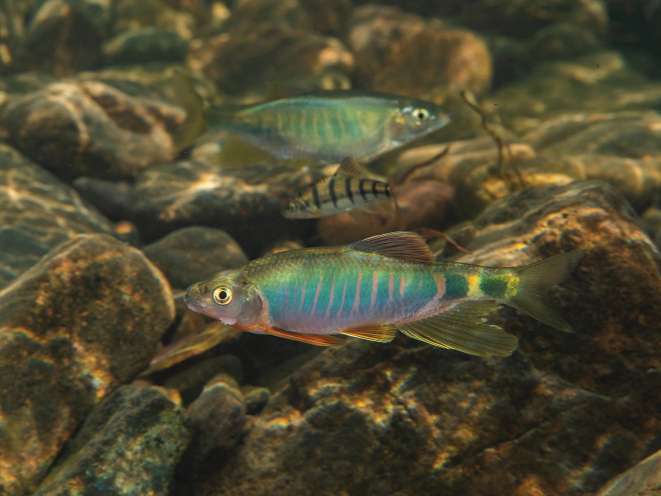
Image of the habitat of *Opsariichthysiridescens* sp. nov., near riverbed with stones.

The new species lives in the headwaters of streams with moderate flow velocities and clear water with small to medium-sized pebbles and boulders in the substrate (Fig. 4).

##### Etymology.

*Iridescens* is the Latin form of the word iridescent. Here, it refers to the unique body color, which is brighter than that of any known species in the genus. In this study, we propose the Chinese common name Hóng Cǎi Mǎ Kǒu Yú (虹彩马口鱼).

### ﻿Morphological analysis

**Figure 5.**
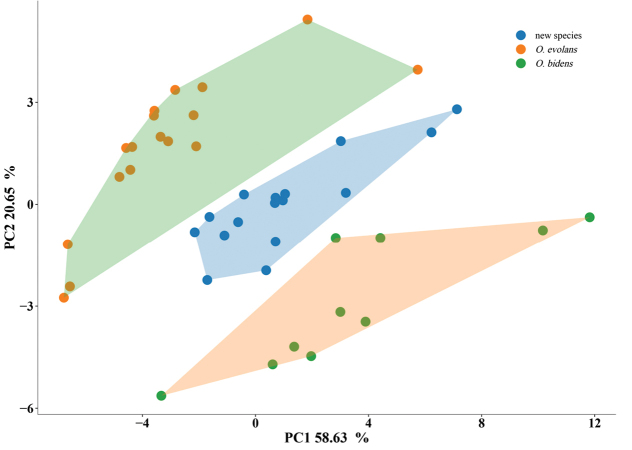
PCA score plots for PC1 and PC2.

PCA was performed on three *Opsariichthys* species based on the morphological data. Fig. 5 shows the principal component score plot. The cumulative contribution of PC1 and PC2 was 79.28%, which represents most of the information in the original data. The contribution rate of PC1 was 58.63%, and the eigenvalue was 23.34, which was the highest contribution to the model. The contribution rate of PC2 was 20.65%, and the eigenvalue was 4.09. In the principal component score plot, *O.evolans* was mainly clustered on the negative side of the origin of the PC1 axis, whereas the new species and *O.bidens* were mainly distributed on the origin of the PC1 axis and on the positive side, so that the three species could be clearly distinguished from each other.

**Figure 6.**
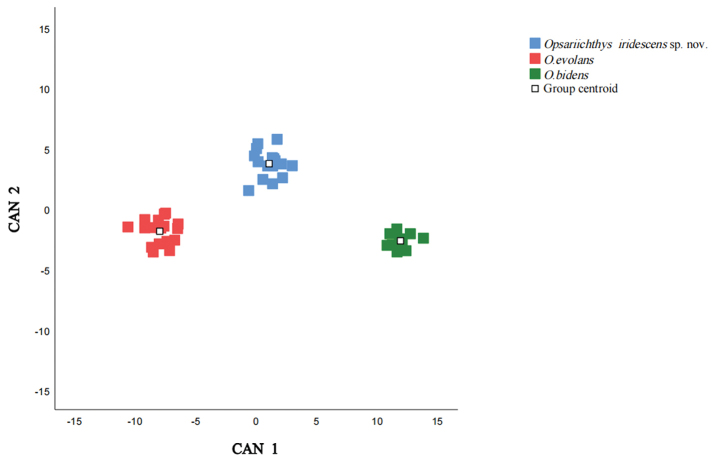
Canonical discriminant score plot for the three species of *Opsariichthys*.

Through typical discriminant analysis, a table of coefficients of typical discriminant functions related to the morphological data was obtained, and two typical discriminant functions were established. The eigenvalues of the two typical discriminant functions were 23.343 and 4.085, and their variance contribution rates were 85.1% and 14.9%, respectively. According to the two discriminant functions, the scores of different *Opsariichthys* species were calculated, and scatter plots of the scores of different *Opsariichthys* species were obtained by using these two discriminant functions as horizontal and vertical coordinates, respectively (Fig. 6). As shown in the scatter plot, none of the three species overlapped, suggesting that they are different species.

### ﻿Molecular phylogenetic analysis

In this study, a total of 72 Cyt *b* gene sequences from 27 species of the opsariichthine group were used, and two additional Cyt *b* sequences from *Aphyocyprischinensis* were used as outgroups. Based on the length heterogeneity of the sequences from GenBank, four sequences were compared to obtain a sequence length of 913 bp for *Z.platypus*, *Opsariichthys* sp. A, *O.acutipinnis*, and *O.duchuunguyeni*, and the remaining 68 opsariichthine group sequences were 1140 bp in length. The base frequencies (excluding outgroups) were A = 25.6%, C = 28.1%, G = 16.2%, and T = 30.1%. The content of A+T (55.7%) was significantly greater than that of G+C (44.3%), which was basically consistent with the characteristics of the mitochondrial genes of fish that have high A and T contents and low G and C contents. There were 672 conserved sites, accounting for 58.9% of the total number of sites; 468 mutated sites, accounting for 41.1% of the total number of sites; 74 single mutated sites, accounting for 6.5% of the total number of sites; and 394 parsimony informative sites, accounting for 34.6% of the total number of sites. The conversion ratio of the sequence was 3.03.

**Figure 7.**
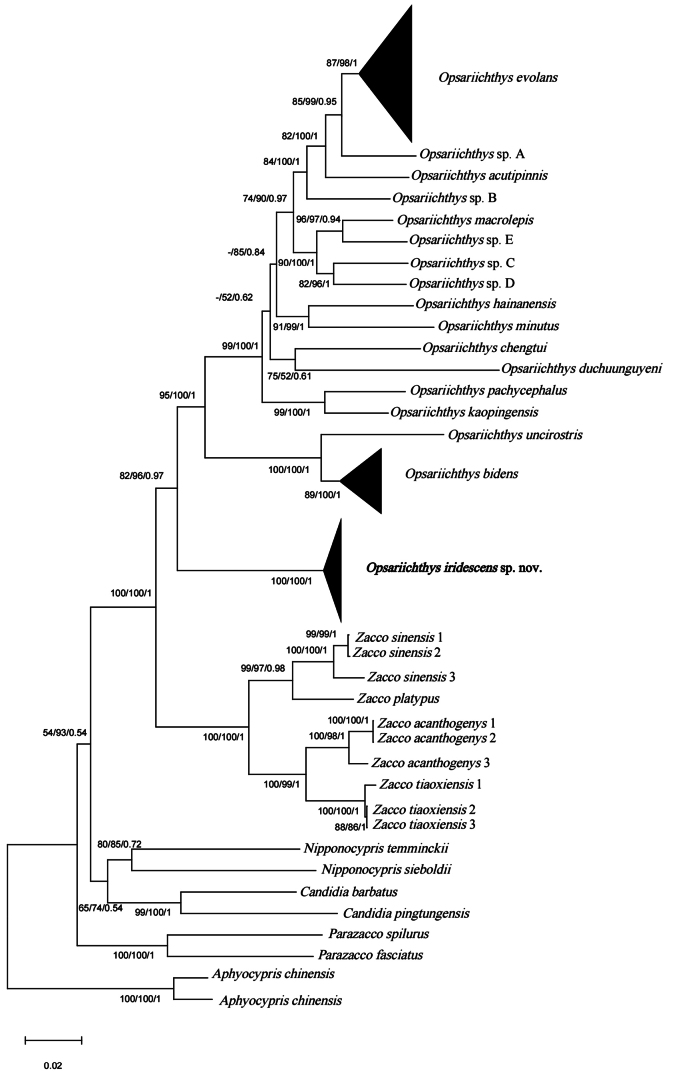
Phylogenetic relationships of opsariichthine derived from the NJ tree based on the Cyt *b* gene sequences; the values at the nodes correspond to the support values for the NJ/ML/BI methods. ‘-’ indicates that the value is less than 50%.

The phylogenetic tree of the opsariichthine group was reconstructed based on the NJ, BI, and ML analyses, and all three trees had a consistent topology despite the differences in support at some branches. Here, we only show the topology of the NJ tree while adding the self-expanding support of the BI and ML trees at the nodes. The topology of the NJ tree (Fig. 7) shows that *Opsariichthys* and *Zacco* form a monophyletic group and are sister groups to each other. The new monophyly species clustered in the *Opsariichthys* group, was located at the base of the genus, and formed a sister group with other *Opsariichthys* species with the support of 82/96/0.97 (NJ/ML/BI).

The genetic distances among the species of the opsariichthine group were calculated based on a K2P model. The genetic distances among the new species and the congeneric species and lineages ranged from 0.143 to 0.186, and those among the inter-genus species ranged from 0.144 to 0.193. Among them, the smallest genetic distance from the new species was observed for *Opsariichthys* sp. D, with a value of 0.143, while the greatest genetic distance from the new species was observed for *Parazaccospilurus*, with a value of 0.193 (Table 5).

**Table 5.** Nucleotide distances between the opsariichthine group species based on the K2P model.

	1	2	3	4	5	6	7	8	9	10	11	12	13	14	15	16	17	18	19	20	21	22	23	24	25	26	27
*O.iridescens* sp. nov.																											
*O.bidens*	0.146																										
*O.evolans*	0.144	0.138																									
*O.acutipinnis*	0.146	0.126	0.055																								
*O.chengtui*	0.150	0.158	0.098	0.110																							
*O.duchuunguyeni*	0.186	0.155	0.128	0.128	0.117																						
*O.hainanensis*	0.152	0.144	0.089	0.109	0.106	0.141																					
*O.kaopingensis*	0.149	0.133	0.092	0.095	0.099	0.144	0.101																				
*O.macrolepis*	0.147	0.135	0.072	0.074	0.093	0.119	0.099	0.087																			
*O.minutus*	0.162	0.154	0.099	0.114	0.109	0.143	0.082	0.105	0.107																		
*O.pachycephalus*	0.144	0.148	0.099	0.115	0.101	0.144	0.106	0.051	0.101	0.094																	
*O.uncirostris*	0.170	0.074	0.156	0.144	0.163	0.184	0.152	0.145	0.148	0.159	0.155																
*Opsariichthys* sp. A	0.155	0.136	0.046	0.060	0.117	0.131	0.100	0.089	0.079	0.110	0.106	0.159															
*Opsariichthys* sp. B	0.147	0.142	0.062	0.061	0.088	0.120	0.097	0.100	0.069	0.100	0.098	0.158	0.068														
*Opsariichthys* sp. C	0.150	0.147	0.077	0.087	0.095	0.121	0.104	0.095	0.059	0.096	0.097	0.159	0.091	0.074													
*Opsariichthys* sp. D	0.143	0.147	0.076	0.091	0.100	0.115	0.097	0.096	0.053	0.103	0.105	0.165	0.092	0.072	0.052												
*Opsariichthys* sp. E	0.153	0.138	0.077	0.088	0.095	0.117	0.102	0.102	0.041	0.104	0.105	0.152	0.094	0.071	0.065	0.070											
*Z.acanthogenys*	0.147	0.164	0.164	0.171	0.164	0.188	0.167	0.160	0.157	0.183	0.162	0.178	0.173	0.166	0.162	0.164	0.170										
*Z.platypus*	0.146	0.154	0.157	0.150	0.156	0.173	0.172	0.155	0.155	0.176	0.166	0.167	0.162	0.165	0.164	0.170	0.172	0.085									
*Z.sinensis*	0.149	0.154	0.156	0.148	0.154	0.173	0.160	0.158	0.143	0.172	0.160	0.169	0.164	0.163	0.167	0.167	0.164	0.076	0.043								
*Z.tiaoxiensis*	0.144	0.164	0.165	0.165	0.163	0.184	0.171	0.152	0.153	0.187	0.162	0.177	0.164	0.170	0.162	0.170	0.168	0.046	0.081	0.080							
*P.fasciatus*	0.192	0.209	0.194	0.202	0.194	0.240	0.183	0.189	0.202	0.216	0.206	0.216	0.195	0.193	0.214	0.195	0.200	0.206	0.198	0.205	0.197						
*P.spilurus*	0.193	0.202	0.204	0.212	0.209	0.254	0.189	0.214	0.198	0.214	0.211	0.221	0.203	0.190	0.212	0.200	0.207	0.196	0.198	0.196	0.195	0.107					
*N.sieboldii*	0.182	0.184	0.192	0.194	0.187	0.221	0.174	0.181	0.188	0.205	0.188	0.192	0.184	0.190	0.192	0.186	0.190	0.168	0.171	0.187	0.172	0.165	0.163				
*N.temminckii*	0.171	0.180	0.186	0.185	0.185	0.207	0.184	0.181	0.191	0.200	0.194	0.190	0.179	0.191	0.192	0.181	0.197	0.161	0.163	0.169	0.161	0.176	0.165	0.126			
*C.barbatus*	0.172	0.196	0.182	0.186	0.181	0.213	0.185	0.194	0.188	0.195	0.198	0.213	0.185	0.166	0.182	0.173	0.193	0.166	0.164	0.171	0.169	0.168	0.163	0.141	0.130		
*C.pingtungensis*	0.187	0.207	0.198	0.204	0.209	0.229	0.209	0.202	0.196	0.218	0.209	0.210	0.200	0.196	0.199	0.183	0.200	0.180	0.172	0.190	0.180	0.178	0.183	0.159	0.154	0.097	

### ﻿Diagnostic key to *Opsariichthys* species

**Table:** 

1	Absence of distinct anterior notches on the upper lip; lateral jaws relatively straight	**2**
–	Presence of a distinct anterior notch on the upper lip; lateral jaws undulated	**9**
2	2 rows of pharyngeal teeth	**3**
−	3 rows of pharyngeal teeth	**5**
3	Fewer than 60 lateral-line scales	**4**
−	More than 60 lateral-line scales	***O.chengtui* (the upper Yangtze River)**
4	2 or 3 rows with 15–21 rather small, rounded tubercles in total that are well separated on the lower jaw in males; body with 11–13 greenish blue stripes of almost equal width in males	***O.macrolepis* (the upper Yangtze River)**
−	Single row of 3–6 rather large, rounded tubercles on the lower jaw of males, united basally to form a plate; 10–13 pale pink strips on the body of males, uniform and narrow on the trunk and widening significantly on the caudal peduncle	***O.iridescens* sp. nov. (southeast China)**
5	Fewer than 42 lateral–line scales; 13 or 14 predorsal scales; 3 scales below the lateral line; very narrow body width; 2 rows with 12–15 rather large and rounded tubercles on the lower jaw in adult males	***O.duchuunguyeni* (northern Vietnam)**
−	More than 42 lateral-line scales; 15–17 predorsal scales; 4 scales below the lateral line modally; a rather narrow to thick body width; a series of 4–7 rounded tubercles on lower jaw in adult males	**6**
6	42–45 lateral-line scales; 15–17 predorsal scales; a rather narrow body width; maxillary that does not extend to or slightly reaches the vertical anterior margin of the orbit; pectoral fin reaching or extending far beyond the origin of the ventral fin	**7**
−	More than 45 lateral-line scales; 18–23 predorsal scales; rather thick body width; maxillary that extends to or far beyond the vertical anterior margin of the orbit; pectoral fin that does not extend beyond the origin of the ventral fin	**8**
7	18–20 circum-peduncular scales; 15 pectoral fin rays modally; maxillary that extends to the vertical anterior margin of the orbit; a pectoral fin that does not extend to the origin of the ventral fin; 9 scales above the lateral line modally	***O.acutipinnis* (southern China)**
−	16 or 17 circum-peduncular scales; 14 pectoral fin rays modally; maxillary that does not extend to the vertical anterior margin of the orbit; pectoral fin that extends far beyond the origin of the ventral fin; 8 scales above the lateral line modally	***O.evolans* (northern Taiwan Island, eastern mainland China)**
8	More than 48 lateral-line scales (mode 50–54); 20–23 predorsal scales; 40 or 41 vertebrae; maxillary that extends to or beyond the vertical midline of the orbit in females; opercle and ventral side of head orange-red to pink-red in adult males	***O.pachycephalus* (northern, middle and western Taiwan Island)**
−	40–45 lateral-line scales (mode 45–47); 18 or 19 predorsal scales; 39 vertebrae; maxillary that extends to or slightly beyond the vertical anterior margin of orbit in females; opercle and ventral side of head bright yellow in adult males	***O.kaopingensis* (southern Taiwan Island)**
9	Fewer than 50 lateral-line scales; 8–10 scales above the lateral line	**10**
−	More than 50 lateral-line scales; 10–12 scales above the lateral line	***O.uncirostris* (Japan and Korea)**
10	45–50 lateral-line scales; rounded tubercles on lower jaw rather small and arranged in 3 rows in males	**11**
−	40–43 lateral-line scales; rounded tubercles on lower jaw rather large and arranged in 2 or 3 rows in males	**13**
11	45–47 lateral-line scales; 8 or 9 scales above lateral line; 17–19 circum-peduncular scales; 40–42 vertebrae	**12**
−	46–50 lateral-line scales; 9 or 10 scales above lateral line; 19 or 20 circum-peduncular scales; 38 or 39 vertebrae	***O.amurensis* (Amur River)**
12	19–21 predorsal scales; 9 scales above lateral line; 41 or 42 vertebrae	***O.bidens* (northern and east China)**
−	16–18 predorsal scales; 8 scales above lateral line modally; 40 or 41 vertebrae	***O.minutus* (southern China)**
13	41–43 lateral-line scales (mode 42); 15 or 16 predorsal scales modally; rounded tubercles large or small arranged in 2 or 3 rows; rather small head; body strongly laterally compressed at position of anal fin origin	14
−	40 or 41 lateral-line scales (mode 41); 17 predorsal scales modally; rounded tubercles on lower jaw rather large and arranged in 2 rows; rather large head; body rather wide at anal fin origin	***O.hainanensis* (Hainan Island)**
14	13–15 pectoral fin rays (mode 14); 16–19 caudal peduncle scales (mode 17); 15–18 predorsal scales (mode 16); 14–16 anterior scales before pelvic origin (mode 15); rounded tubercles on lower jaw rather large and arranged in 3 rows in males; body with 14 greenish blue cross-bars in males; maxillary that extends to vertical midline of orbit in females; snout length of approximately 32–33% in males; interorbital width of approximately 30% in males	***O.dienbienensis* (northern Vietnam)**
−	13 or 14 pectoral fin rays (mode 13); 18 caudal peduncle scales modally; 15–18 predorsal scales (mode 15); 13–15 anterior scales before the pelvic origin (mode 14); rounded tubercles on lower jaw rather small and arranged in 2 or 3 rows in males; body with 13 greenish blue cross-bars in males; maxillary does not extend to vertical midline of orbit in females; snout length of approximately 30% in males; interorbital width of approximately 27–28% in males	***O.songmaensis* (Ma River of Vietnam)**

## ﻿Discussion

For a long time, the genus *Opsariichthys* was thought to include only one species, *O.bidens*, which was widely distributed in East Asia (Chen 1982; Chen and Chu 1998). With the help of modern molecular techniques, the nuptial tubercles on the cheeks of males and the lateral cross-bars on the body, which were first identified by Chen et al. (2009) and later confirmed by Huang et al. (2017), were found to be the key diagnostic features distinguishing this genus from its sister genus *Zacco*. Thus, the taxonomy of the two genera gradually became clearer. Therefore, based on the stripe features and phylogeny of this study, we ascribe *O.iridescens* sp. nov. to the genus *Opsariichthys*. In addition, based on geographic distribution, the results of morphological and PCA analyses also indicated the validity of the new species. With many independent rivers and diverse habitats, southeast mainland China is rich in freshwater fish species, and some species have a common distribution with northern Taiwan, including *O.evolans*, which was used for the comparison in this study. According to our investigations, in addition to the new species, only *O.bidens* and *O.evolans* are found in Zhejiang Province, north of the Wuyi Mountains, and are distinct from the other congeners. Based on our observations, all *Opsariichthys* species can be divided into two types: one with a large mouth, a conspicuous anterior notch on the upper lip, and undulated jaws; and another with a small mouth, no distinct anterior notch on the upper lip, and relatively straight jaws. These two types are morphological adaptations to feeding habits, carnivores and omnivores, respectively. The new species belongs to the latter; however, the phylogenetic analysis in this study shows that the two types do not form monophyletic groups, indicating that mouth shape may be a derived evolutionary trait. In addition to the difference in scale numbers (Table 3), the new species also has several obvious morphological features that distinguish it from other species in the same group: 1) the nuptial tubercles on the cheeks and lower jaw of the adult males were united basally to form a plate, similar to the species of *Zacco*; 2) two rows of pharyngeal teeth; and 3) the narrow, pale, lateral cross-bars that are almost uniform in width on the trunk and widening significantly on the caudal peduncle (Table 4). In the diagnostic key for *Opsariichthys* species presented above the data for all but three species was obtained from published sources (Yang and Huang 1964; Chen et al. 2009; Huynh and Chen 2013; Wang et al. 2019).

Wang et al. (2019) used a 3% Cyt *b* gene genetic distance to delimit the opsariichthine fish species, identified 20 haplogroups of *Opsariichthys*, and reported that the species diversity of this genus was underestimated. The new species reported herein does not belong to any of the haplogroups in Wang et al. (2019), and its genetic distance from both congeneric and intergeneric species exceeds 14%, which is much greater than their 3% limit (see Table 5). Our phylogenetic results are consistent with the results of previous studies (Huang et al. 2017; Wang et al. 2019; Zhang et al. 2023) (Fig. 7). *Opsariichthys* and *Zacco*, which both have lateral cross-bars, are both monophyletic and form separate clades, which supports the use of stripes as key diagnostic features for distinguishing them. Moreover, *O.iridescens* sp. nov., as a monophyletic group, is located at the base of all *Opsariichthys* species. In conclusion, the genetic distance and phylogenetic analyses support morphological distinctiveness and the validity of the new species.

### ﻿Comparative materials

*O.evolans*: SHOU2021060004-006, 3 specimens, 67.5–112.0 mm SL, She County, Qiantang River System, Anhui Province, China; SHOU2021060091, 1 specimen, 103.3 mm SL, Suichang County, Qiantang River System, Zhejiang Province, China; SHOU2021060145, 1 specimen, 83.5 mm SL, Qingyuan County, Ou River System, Zhejiang Province, China; SHOU202208005-006, 2 specimens, 71.4–77.5 mm SL, Shangyu District, Cao’e River System, Zhejiang Province, China; SHOU202208031-040, 10 specimens, 71.2–84.0 mm SL, Shengzhou City, Cao’e River System, Zhejiang Province, China.

*O.bidens*: SHOU202209008-012, 5 specimens, 76.1–100.6 mm SL, Qingyuan County, Ou River System, Zhejiang Province, China; SHOU202111001-005, 5 specimens, 99.6–131.6 mm SL, Shengzhou City, Cao’e River System, Zhejiang Province, China.

## Supplementary Material

XML Treatment for Opsariichthys iridescens

## ﻿Additional information

### Author ORCIDs

Jia-Jun Zhou https://orcid.org/0000-0003-1038-1540

Hong-Di Gao https://orcid.org/0009-0004-6891-3209

Jin-Quan Yang https://orcid.org/0000-0003-0387-1824

### Data availability

All of the data that support the findings of this study are available in the main text.
